# The reproducibility and predictivity of radiomic features extracted from dynamic contrast-enhanced computed tomography of hepatocellular carcinoma

**DOI:** 10.1371/journal.pone.0310486

**Published:** 2024-09-13

**Authors:** Abdalla Ibrahim, Siddharth Guha, Lin Lu, Pengfei Geng, Qian Wu, Yen Chou, Hao Yang, Delin Wang, Lawrence H. Schwartz, Chuan-miao Xie, Binsheng Zhao

**Affiliations:** 1 Department of Radiology, Memorial Sloan Kettering Cancer Center, New York, New York, United States of America; 2 Department of Radiology, Columbia University Irving Medical Center, New York, New York, United States of America; 3 First Affiliated Hospital of Nanjing Medical University, Jiangsu, China; 4 Department of Medical Imaging, Fu Jen Catholic University Hospital, New Taipei City, Taiwan; 5 Department of Radiology, Sun Yat-Sen University Cancer Center, Guangzhou, China; University of Pisa, ITALY

## Abstract

**Purpose:**

To assess the reproducibility of radiomic features (RFs) extracted from dynamic contrast-enhanced computed tomography (DCE-CT) scans of patients diagnosed with hepatocellular carcinoma (HCC) with regards to inter-observer variability and acquisition timing after contrast injection. The predictive ability of reproducible RFs for differentiating between the degrees of HCC differentiation is also investigated.

**Methods:**

We analyzed a set of DCE-CT scans of 39 patients diagnosed with HCC. Two radiologists independently segmented the scans, and RFs were extracted from each sequence of the DCE-CT scans. The same lesion was segmented across the DCE-CT sequences of each patient’s scan. From each lesion, 127 commonly used RFs were extracted. The reproducibility of RFs was assessed with regard to (i) inter-observer variability, by evaluating the reproducibility of RFs between the two radiologists; and (ii) timing of acquisition following contrast injection (inter- and intra-imaging phase). The reproducibility of RFs was assessed using the concordance correlation coefficient (CCC), with a cut-off value of 0.90. Reproducible RFs were used for building XGBoost classification models for the differentiation of HCC differentiation.

**Results:**

Inter-observer analyses across the different contrast-enhancement phases showed that the number of reproducible RFs was 29 (22.8%), 52 (40.9%), and 36 (28.3%) for the non-contrast enhanced, late arterial, and portal venous phases, respectively. Intra- and inter-sequence analyses revealed that the number of reproducible RFs ranged between 1 (0.8%) and 47 (37%), inversely related with time interval between the sequences. XGBoost algorithms built using reproducible RFs in each phase were found to be high predictive ability of the degree of HCC tumor differentiation.

**Conclusions:**

The reproducibility of many RFs was significantly impacted by inter-observer variability, and a larger number of RFs were impacted by the difference in the time of acquisition after contrast injection. Our findings highlight the need for quality assessment to ensure that scans are analyzed in the same physiologic imaging phase in quantitative imaging studies, or that phase-wide reproducible RFs are selected. Overall, the study emphasizes the importance of reproducibility and quality control when using RFs as biomarkers for clinical applications.

## Introduction

Radiomics is an emerging translational field that aims to extract and analyze data from medical images, using quantitative image features known as radiomic features (RFs), to support evidence-based clinical decision-making [1–3]. Machine learning models built from RFs have a wide range of clinical applications, including predicting cancer prognosis and predicting aortic dissection [4, 5]. However, for these models to have greater legitimacy in clinical practice, the RFs from which they are built must be reproducible under a wide variety of factors related to image acquisition and processing [6–9]. For instance, a feature that is meaningful in one dataset may not be in another due to its sensitivity to acquisition settings (e.g., scanner manufacturer, scanning technique, and reconstruction parameters). As a result, the reproducibility of RFs has been extensively studied using human cohorts of a variety of pathologies and phantom data as well [10–18].

Despite this, limited studies have examined the reproducibility of RFs across and within different CT contrast-enhancement phases, mainly due to the lack of dynamic contrast-enhanced computed tomography (DCE-CT) images for this purpose. DCE-CT is typically used in the diagnosis and characterization of primary liver lesions, such as hepatocellular carcinoma [19–21]. DCE-CT scans are taken at different time points as the contrast travels through various organs and clinically be classified into three contrast enhancement phases (arterial, portal venous, and delayed phase) based on the LI-RADS 2018 criteria [22]. Because of the sensitivity of RFs, the accuracy and validity of models built using these features extracted from CT images acquired in different phases can be impacted [23–25]. There exists a need to study their reproducibility across and within all the contrast enhancement phases. However, there is a scarcity of literature on this topic, particularly in the imaging of liver lesions. Since Hepatocellular Cell Carcinoma (HCC) lesions show different characteristics in different imaging phases [26], biologically meaningful RFs could potentially have unique measurement values across the different phases [15, 27, 28]. However, to date, no study has evaluated the reproducibility of RFs within time windows in each contrast enhancement phase.

In this study, we present a unique dataset of DCE-CT scans from HCC patients. Our aims are: (i) to investigate the effects of differences in lesion segmentation on the reproducibility of RFs, and (ii) to assess the reproducibility of RFs within and across contrast enhancement phases, namely the non-contrast enhanced (NCE), late arterial (L-AP), and portal venous phases (PVP). Ultimately, the goal is to guide robust analysis of RFs extracted from contrast enhanced CTs.

## Materials and methods

### Patient data

**Fig 1:**
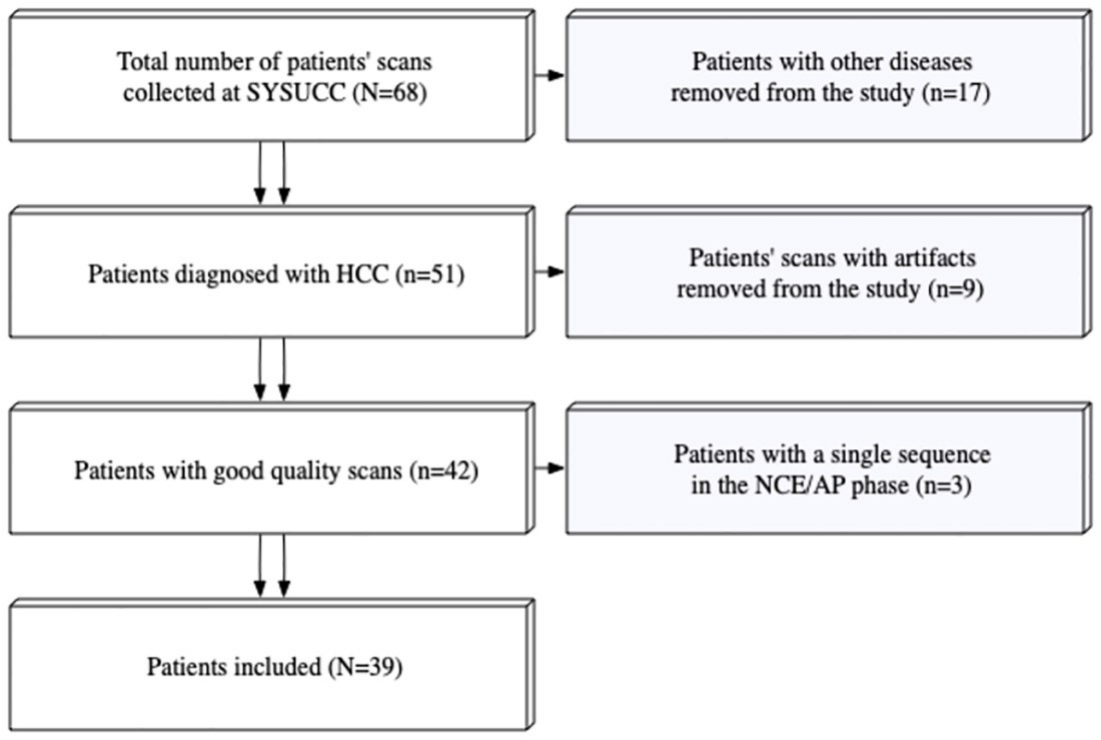
A flowchart of the patients’ inclusion process.

Completely de-identified DCE-CT scans of 68 patients who underwent liver lesion assessment were retrospectively collected at a single medical center, with the institutional review board approval. The inclusion criteria were: (i) Pathologically proven HCC; (ii) Absence of artifacts in the scans. Patients with pathologic diagnoses other than HCC (n = 17), patients with scans containing artifacts (n = 9), and those with missing sequences (n = 3) were excluded. This resulted in a total of 39 patients included for analysis in this study (Fig 1). All Scans were acquired prior to the start of treatment. The study was conducted in accordance with the Declaration of Helsinki and was approved by the Institutional Review Board of Sun Yat-Sen University Cancer Center (SYSUCC) (protocol code 510060, approved on November 9th, 2022). Informed consent was waived by the Institutional Review Board of SYSUCC. The data was accessed for research purposes on the 11^th^ of January 2023.

### Imaging data, segmentation, and RFs extraction

The DCE-CT scans were acquired using a TOSHIBA Aquilion scanner. Each sequence scan included four slices, with time intervals between consecutive DCE sequences of 1–2 seconds. Scanning of the patients commenced immediately after contrast injection. The number of DCE-CT sequences per patient ranged between 36 and 42 sequences. The acquisition and reconstruction parameters for the included DCE-CT scans are presented in Table 1.

**Table 1:** Acquisition and reconstruction parameters of the imaging data.

Vendors	Model	X-Ray tube Current (mA)	Exposure time (ms)	kVP	Reconstruction kernel	Slice thickness (mm)	Pixel spacing (mm^2^)
Toshiba	Aquilion	50–250	500–4000	120	FC02	2, 5, 8	0.56x0.56–1.0x1.0
					FC04		
					FL03		

The volumes of interest (VOIs) of HCCs were independently segmented by two radiologists (QW and PG, with four and five years of experience in abdominal imaging, respectively) using an integrated tumor segmentation tool customized from the open-source Weasis platform [29]. Each radiologist segmented the VOIs on the sequence where the tumor was most visible. The segmentations were then automatically propagated to the remaining sequences, and further reviewed by each radiologist to ensure correct and consistent lesion segmentation across all sequences. RFs were extracted from the VOIs using an in-house software. A set 127 RFs was extracted from each lesion, derived from different feature classes, including ‘First Order Statistics’, ‘Sigmoid Feature’, ‘Discrete Wavelet Transform’, ‘Edge Frequency’, ‘Fractal Dimension’, ‘GTDM’, ‘Gabor’, ‘LAW filter’, ‘LOG feature’, ‘Run Length’, ‘Spatial correlation’, ‘GLCM’, to characterize image patterns as comprehensively as possible. More details about feature class definitions as well as implementation details can be found in our previous work [10]. No image resampling was performed, and RFs were extracted by setting the bin width to 25 Hounsfield units. The extracted RFs are provided in S1 File.

### Analysis of inter-observer variability

**Fig 2:**
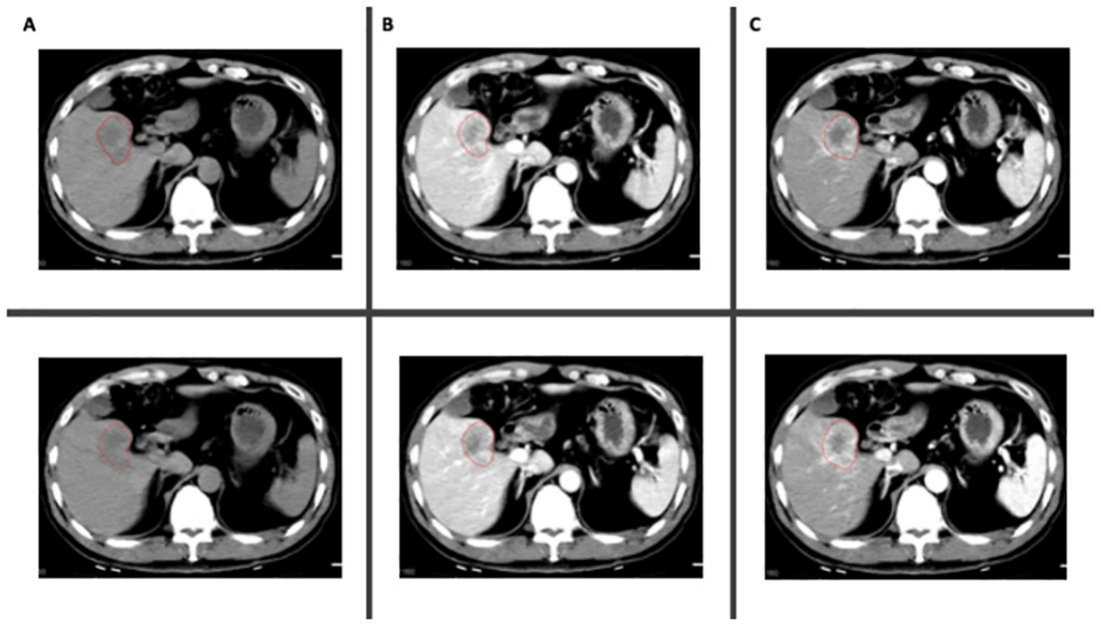
Example of segmentations in two different sequences per phase; (A) NCE; (B) L-AP; (C) PVP.

Two radiologists (QW and PG) independently assigned one of the labels (NCE, L-AP, or PVP) to each of the DCE sequences and segmented the HCC lesions (Fig 2). The labels were based on the LI-RADS Version 2018 criteria for defining dynamic CT phases, as well as other commonly used clinical criteria [22, 30]. Disagreements over the labels were reviewed and discussed with a third radiologist (YC, with six years of experience), and a consensus was reached on all labels. The similarity in the segmentations between the two radiologists was assessed using Dice Similarity Coefficient (DSC) [31]. The agreement in feature values extracted from all the sequences between the radiologists’ segmentations was assessed as one of the primary endpoints.

### Analysis of effects of phase variability

**Fig 3:**
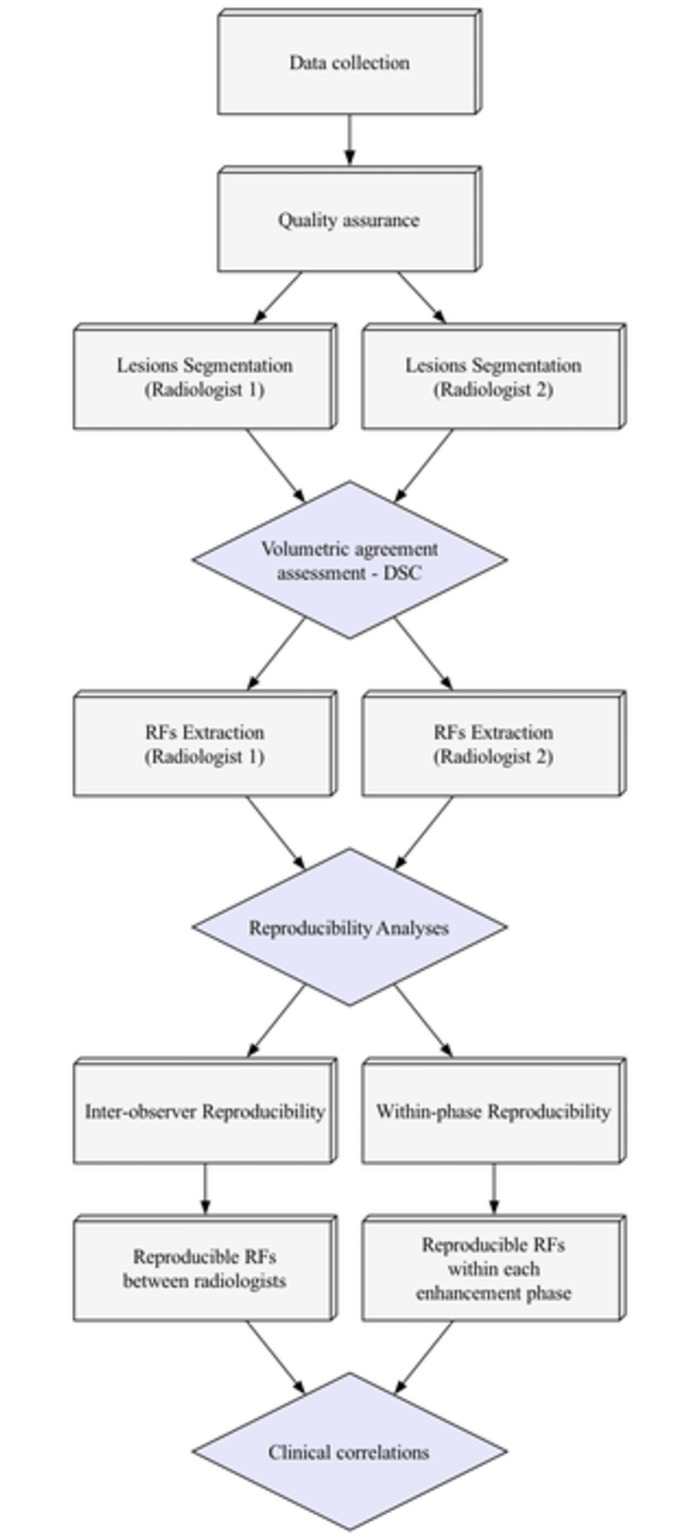
Study workflow.

To assess the agreement in RF values within different phases for each radiologist independently, a different approach was used. The number of sequences within each phase needed to be the same for all the patients. Therefore, the fewest number of sequences available per phase across all patients was identified and set as the number of sequences to be included. Several patients had only two NCE sequences, therefore only the first two NCE sequences were included for all the patients. For the L-AP phase, seven sequences were selected for each patient: the first two, the middle three, and the last two sequences. Similarly, seven PVP sequences were included for each patient: the first two, the middle three, and the last two sequences. Pairwise comparisons were performed across all 16 selected sequences. The concordance correlation coefficient (CCC) with a cutoff of 0.90 was used to identify the within-phase reproducible RFs. Following the identification of reproducible RFs, features that were found to be highly correlated were removed. High correlation was defined as Spearman’s R > 0.90. When two RFs were found to be highly correlated, the one with the higher average correlation with the remaining RFs was removed. The study workflow is depicted in Fig 3.

### Statistical analyses

All statistical analyses were performed using R language on RStudio version 2022.02.0 [32, 33]. To assess the agreement in RF values between the two radiologists, the CCC was used [34]. Pairwise comparisons were made across the included scans. RF values extracted from all segmented lesions by the two radiologists were compared once using all the data, and once within each contrast enhancement phase. RFs with CCC values equal to or higher than 0.90 were considered reproducible [35].

To assess the correlations between the reproducible RFs and the degree of histologic differentiation of the HCC lesions, Wilcoxon rank-sum test to assess differences in values across groups of well to moderately differentiated tumors and moderately to poorly differentiated tumors was used. The significance level was set at 0.05.

Machine learning was used to develop classification models using the reproducible RFs. For this analysis, the data were first split into 29 (74%) training and 10 (26%) testing sets. The outcomes in the training set were balanced using the synthetic minority oversampling technique (SMOTE). Following that, if the number of the reproducible features was less than 3, all were used for building the final model. If the number of reproducible RFs exceeded 3, recursive feature elimination with treebag functions and 5-fold cross-validation was used to select the most important RFs, with a maximum of 3. XGBoost algorithm was used to develop the classification model. The model was validated on the test set, and the AUC, sensitivity, specificity, negative predictive value (NPV), and positive predictive value (PPV) were used to assess the models’ performance.

## Results

### Patient data

The patients included (N = 39) had a median (IQR) age of 57 (43, 66) years, and 35 (89.7%) were male. Of the 39 patients, 37 (94.9%) had chronic Hepatitis B infection, and 7 (17.9%) reported consuming a minimum of 100 ml per day for longer than 30 years. According to pathology assessment, 3 (7.7%) patients had well-differentiated HCC, 4 (10.3%) had well to moderately differentiated HCC, 16 (41.0%) had moderately differentiated HCC, 12 (30.8%) had moderately to poorly differentiated HCC, and 4 (10.3%) had poorly differentiated HCC. The tumors had an average volume (± standard deviation) of 18537.6 (±16548.5) mm^3^. The average largest tumor diameter was 33.7 (±13.1) mm. All tumors were best visualized and first segmented on the L-AP sequences.

### Inter-observer variability

The assessment of segmentation similarity between the radiologists showed a DSC of 0.79. Among the extracted features, 29 (22.8%) RFs were concordant across the NCE sequences; 52 (40.9%) RFs were concordant across the L-AP sequences; and 36 (28.3%) RFs were concordant across the portal venous phase sequences (Table 2).

**Table 2:** List of non-correlated reproducible RFs within the different phases between the radiologists.

NCE	L-AP	PVP
GLCM Contrast	GLCM Contrast	GLCM Sum Squares
GLCM Sum Squares	LoG Z Uniformity	GLCM Contrast
GTDM Strength	GLCM Correlation	LoG Z Uniformity
Intensity 75percent	GLCM Sum Squares	GLCM ASM
Intensity Median	Gabor Min Z	RSRLGL Emphasis
Intensity Energy	Intensity 25percent	Gabor Min Z
Intensity 25percent	Intensity Root mean square	GLCM Correlation
RSRLGL Emphasis	Intensity Mean	RHGLR Emphasis
Intensity Root mean square	Intensity Median	RGL Uniformity
Intensity Mean	Intensity Energy	RSR Emphasis
RGL Uniformity	Intensity 75percent	Sigmoid Amplitude Mean
Intensity PeakPosition	Sigmoid Offset Mean	Gabor Median Z
RHGLR Emphasis	GLCM ASM	Gabor Min Z Boundary
RSR Emphasis	Intensity Maximum	Intensity 75percent
Sigmoid Amplitude Mean	Intensity PeakPosition	Intensity Energy
GLCM Correlation	Gabor sum Z	Gabor Mean Z Boundary
Gabor Max Z	RSRLGL Emphasis	Intensity Median
Intensity Uniformity	RHGLR Emphasis	Intensity Mean
GLCM ASM	GLCM Sum Entropy	Gabor Median Z Boundary
Intensity Mean absolute deviation	GLCM Entropy	Intensity Root mean square
DWF Z LLL	Gabor Mean Z Boundary	GTDM Strength
Intensity Variance	Laws 3 Z NoBoundary	Intensity 25percent
DWF Z LL	Gabor Median Z Boundary	Intensity PeakPosition
Spatial Correlation	GLCM Cluster Tendency	Gabor Max Z
DWF Z L	Gabor Mean Z	GTDM Complexity
LoG Z Uniformity	GLCM IDM	Gabor sum Z
Intensity Std	Sigmoid Amplitude Mean	Gabor Mean Z
Gabor Min Z	Sigmoid Amplitude Std	Intensity Uniformity
Intensity Entropy	Sigmoid Offset Mean	GLCM Diff Entropy
	Sigmoid Offset Std	Intensity Entropy
	Spatial Correlation	Intensity Mean absolute deviation
	RSRHGL Emphasis	Emphasis
	Intensity Entropy	DWF Z LLL
	Intensity Mean absolute deviation	Intensity Std
	Sigmoid Amplitude Mean	DWF Z LL
	RLRHGL Emphasis	Laws 6 Z Boundary
	Intensity Uniformity	RLRHGL Emphasis
	Intensity Std	
	Intensity Variance	
	GLCM Diff Entropy	
	RLGLR Emphasis	
	GLCM MCC	
	DWF Z LLL	
	RLR Emphasis	
	GLCM IMC2	
	GLCM Homogeneity	
	DWF Z LL	
	RPL Uniformity	
	DWF Z L	
	GLCM Max Prob	
	RGL Uniformity	
	GLCM IMC1	
	GLCM Diff Variance	
	RSR Emphasis	
	Gabor Median Z	
	Laws 4 Z NoBoundary	
	GLCM Cluster Tendency	

RSRLGL: Run Short Run Low Gray Level; RGL: Run Gray Level; RSR: Run Short Run; RSRLGL: Run Short Run Low Gray Level; RLRHGL: Run Long Run High Gray Level; RHGLR: Run High Gray Level Run; RLRLGL: Run Long Run Low Gray Level; RLGLR: Run Low Gray Level Run; RPL: Run Primitive Length; RLR: Run Long Run; RSR: Run Short Run; RGL: Run Gray Level.

### Effects of phase variability

#### Inter-phase variability

**Fig 4:**
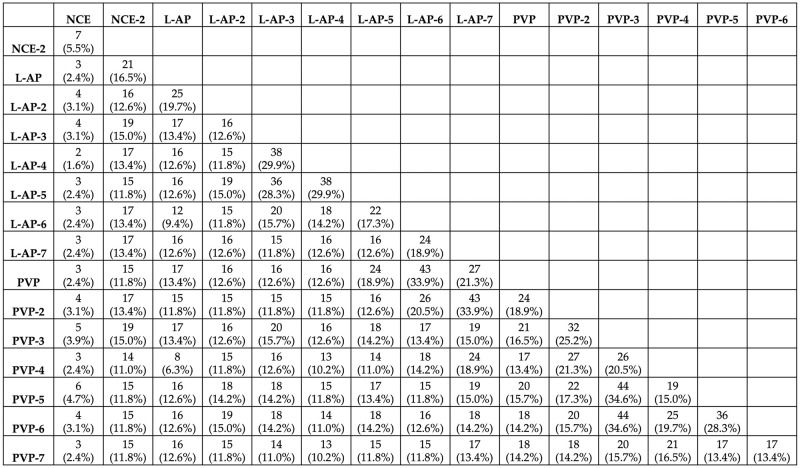
The number of reproducible RFs across the different pairs for radiologist 1.

For Radiologist 1, the number of reproducible RFs varied across the pairwise comparisons, ranging from 2 (1.6%) to 44 (34.6%), with a median of 16 (12.6%) reproducible RFs (Fig 4).

**Fig 5:**
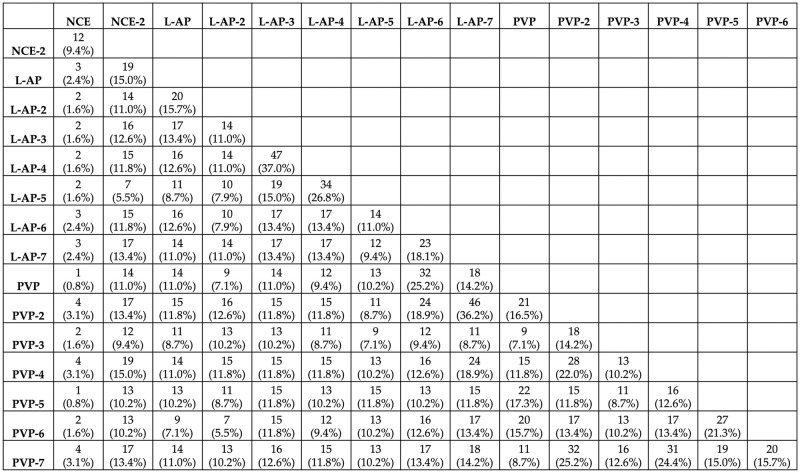
The number of reproducible RFs across the different pairs for radiologist 2.

For Radiologist 2, the number of reproducible RFs varied across the pairwise, ranging from 1 (0.8%) to 47 (37%), with a median of 14 (11%) reproducible RFs (Fig 5).

#### Intra-phase reproducible features

For Radiologist 1, the number of reproducible RFs in the NCE sequence comparisons was 7 (5.5%). For the L-AP and PVP sequences, 9 (7.1%), and 15 (11.8%) were found to be reproducible, respectively (Table 3).

**Table 3:** Reproducible RFs within each phase for radiologist 1.

NCE	L-AP	PVP
RPL Uniformity	Gabor sum Z	DWF Z L
LoG Z Uniformity	Gabor Max Z	DWF Z LL
LoG Z Entropy	Gabor Mean Z	DWF Z LLL
Laws 1 Z Boundary	Gabor sum Z Boundary	Gabor sum Z
Laws 1 Z NoBoundary	Gabor Max Z Boundary	Gabor Max Z
GLCM Cluster Tendency	Gabor Mean Z Boundary	Gabor Mean Z
GLCM Sum Squares	Gabor Median Z Boundary	Gabor Median Z
	LoG Z MGI	Gabor sum Z Boundary
	LoG Z Entropy	Gabor Max Z Boundary
		Gabor Mean Z Boundary
		Gabor Median Z Boundary
		LoG Z MGI
		LoG Z Entropy
		LoG Z Uniformity
		RPL Uniformity

For Radiologist 2, the number of reproducible RFs in the NCE sequences comparison was 12 (9.4%). For L-AP, and PVP sequences, 5 (3.9%), and 10 (7.9%), RFs were found to be reproducible across all comparisons, respectively (Table 4).

**Table 4:** Reproducible RFs within each phase for radiologist 2.

NCE	L-AP	PVP
RPL Uniformity	Gabor_Median_Z	Gabor sum Z
LoG Z Uniformity	Gabor_Median_Z_Boundary_	Gabor Mean Z
LoG Z Entropy	LoG_Z_MGI	Gabor Median Z
Laws 1 Z Boundary	LoG_Z_Entropy	Gabor sum Z Boundary
GLCM Cluster Tendency	LoG_Z_Uniformity	Gabor Mean Z Boundary
GLCM Sum Squares		Gabor Median Z Boundary
Laws 2 Z Boundary		LoG Z MGI
Laws 1 Z NoBoundary		LoG Z Entropy
Spatial Correlation		LoG Z Uniformity
Laws 2 Z NoBoundary		Gabor sum Z
Intensity 75percent		
GTDM Complexity		

### Clinical correlations

#### Descriptive statistics

The association between the reproducible RFs and the degree of histologic differentiation of HCC was assessed for each reproducible RF within each phase per radiologist.

The descriptive statistics of reproducible RFs, and Wilcoxon’s p value for radiologists 1 and 2 are presented in Tables 5 and 6, respectively.

**Table 5:** Descriptive statistics of reproducible RFs for radiologist 1.

		Well/ well to moderately differentiated		Moderately/moderately to poorly differentiated		Wilcoxon’s P
Feature	Reproducible in	Mean	Std	Mean	Std	
LoG Z Entropy	All	0.81084088	0.19784667	0.73873998	0.19999369	0.02
LoG Z Uniformity	All	0.78372545	0.06505319	0.80779936	0.06743802	0.007
RPL Uniformity	All	5711.28719	3601.99597	7096.44203	5113.26691	0.19
GLCM Cluster Tendency	NCE	4.13538221	2.57371024	25.3603648	80.6924916	0.04
GLCM Sum Squares	NCE	1.21385	0.68590361	6.51011919	20.2192168	0.06
Laws 1 Z Boundary	NCE	-5.4428589	30.4929671	75.1900693	238.403838	0.04
Laws 1 Z NoBoundary	NCE	-0.0012624	29.967227	56.6860394	194.590942	0.21
Gabor Max Z	L-AP, PVP	191738.865	89148.508	165261.087	77492.9668	0.02
Gabor Max Z Boundary	L-AP, PVP	142946.827	72493.964	124979.013	63201.4264	0.07
Gabor Mean Z	L-AP, PVP	102997.378	43414.2068	92292.0788	42779.8162	0.03
Gabor Mean Z Boundary	L-AP, PVP	73125.5601	33650.0336	65258.6635	32660.1312	0.06
Gabor Median Z Boundary	L-AP, PVP	68511.5623	29154.9881	62058.8812	31611.4923	0.04
Gabor sum Z	L-AP, PVP	102997.378	43414.2068	92292.0788	42779.8162	0.03
Gabor sum Z Boundary	L-AP, PVP	73125.5601	33650.0336	65258.6635	32660.1312	0.06
LoG Z MGI	L-AP, PVP	-106.45322	45.3658445	-97.463615	43.207301	0.19
DWF Z L	PVP	46760.9049	11789.8323	50914.6943	12912.3405	0.03
DWF Z LL	PVP	182570.699	47222.3263	199565.35	52601.3594	0.04
DWF Z LLL	PVP	696397.637	189172.778	766498.316	215973.343	0.04
Gabor Median Z	PVP	98430.8248	42631.8654	91752.466	43439.3522	0.18
LoG Z Entropy	All	0.81084088	0.19784667	0.73873998	0.19999369	0.02
LoG Z Uniformity	All	0.78372545	0.06505319	0.80779936	0.06743802	0.007

**Table 6:** Descriptive statistics of reproducible RFs for radiologist 2.

		Well/ well to moderately differentiated		Moderately/moderately to poorly differentiated		Wilcoxon’s P
Feature	Reproducible in	Mean	Std	Mean	Std	
LoG Z Entropy	All	0.84017141	0.20049751	0.77365664	0.21928965	0.05
LoG Z Uniformity	All	0.7740279	0.06662804	0.79548159	0.07492333	0.03
RPL Uniformity	NCE	5313.05116	3068.62499	6447.44889	4782.4508	0.63
Laws 1 Z Boundary	NCE	-1.5328711	32.3471104	93.3039239	284.303348	0.11
GLCM Cluster Tendency	NCE	3.68511314	2.09531987	32.0283901	92.7285088	0.04
GLCM Sum Squares	NCE	1.08411679	0.59919914	8.18591084	23.2482303	0.05
Laws 2 Z Boundary	NCE	-0.3968899	5.82649192	4.96820483	20.4548899	0.87
Laws 1 Z NoBoundary	NCE	0.01004171	26.1001051	65.1889458	226.154652	0.27
Spatial Correlation	NCE	3.9978685	0.00285726	3.99639061	0.01241057	0.23
Laws 2 Z NoBoundary	NCE	-0.0051824	3.36182758	3.57497103	13.4274582	0.81
Intensity 75percent	NCE	46.0714286	9.96504881	37.765625	8.4303725	0.002
GTDM Complexity	NCE	22.7597394	17.2464581	99.6954943	183.20409	0.13
Gabor Median Z	L-AP, PVP	95078.2523	38553.17	92149.0064	45708.9279	0.21
Gabor Median Z Boundary	L-AP, PVP	67950.499	28335.9102	65638.1497	33849.1774	0.21
LoG Z MGI	L-AP, PVP	-111.50057	45.563221	-104.89081	48.0902486	0.19
Gabor sum Z	PVP	110196.985	49865.3778	103158.139	46503.8447	0.31
Gabor Mean Z	PVP	110196.985	49865.3778	103158.139	46503.8447	0.31
Gabor sum Z Boundary	PVP	78687.0126	38459.4449	72504.6692	34873.3984	0.22
Gabor Mean Z Boundary	PVP	78687.0126	38459.4449	72504.6692	34873.3984	0.22
LoG Z Entropy	All	0.84017141	0.20049751	0.77365664	0.21928965	0.04

#### Classification models: NCE

**Fig 6:**
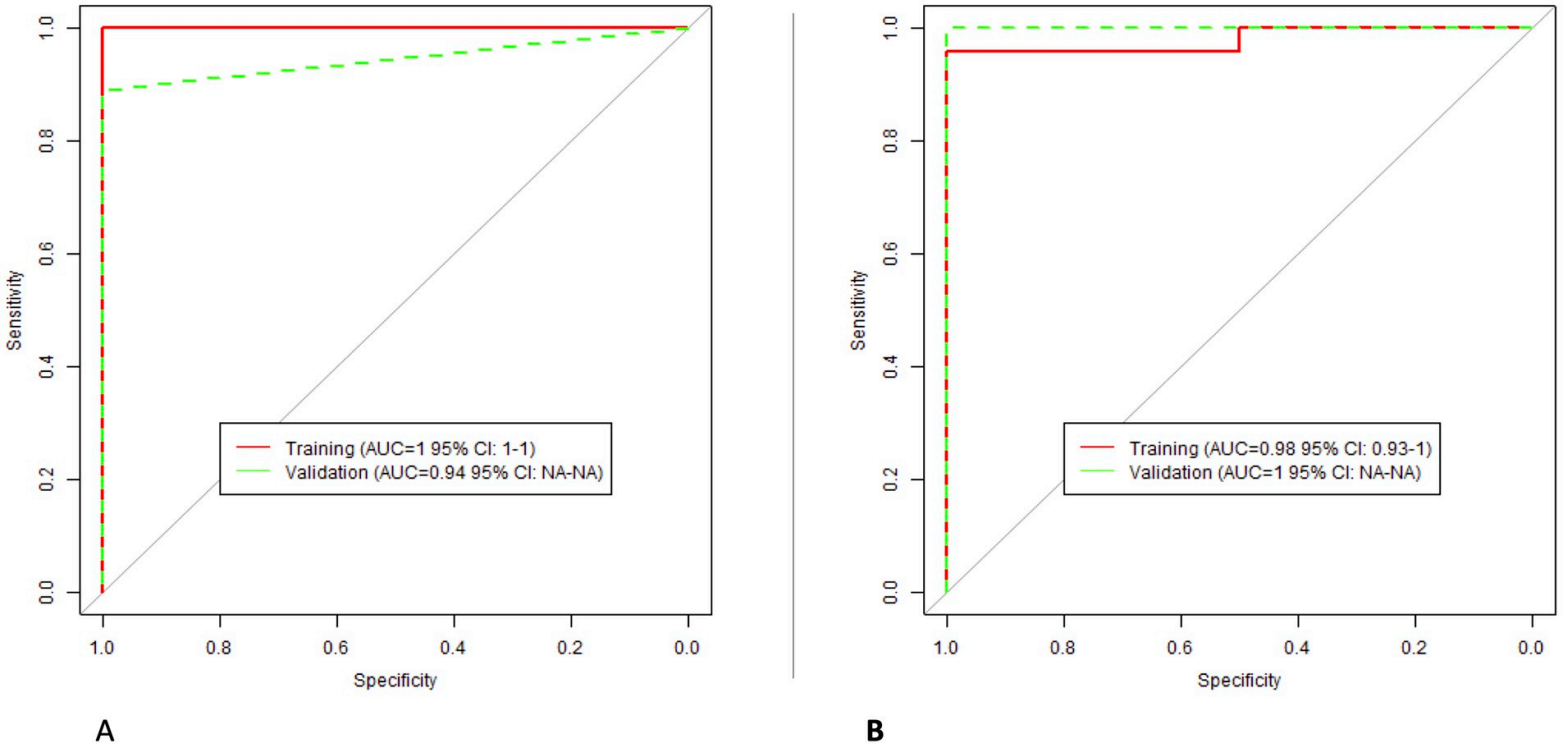
The performance of NCE classification models; (A) Radiologist 1; (B) Radiologist 2.

XGBoost algorithms to classify the degree of HCC lesion differentiation were built using the reproducible RFs. For radiologist 1, the selected RFs were: “GLCM Sum Squares”, “GLCM Cluster Tendency”, and “Laws 1 Z Boundary”. For radiologist 2, the selected features were: “Intensity 75percent”, “Spatial Correlation”, and “GLCM Cluster Tendency”. The models’ performance is presented in Fig 6.

#### Classification models: L-AP

**Fig 7:**
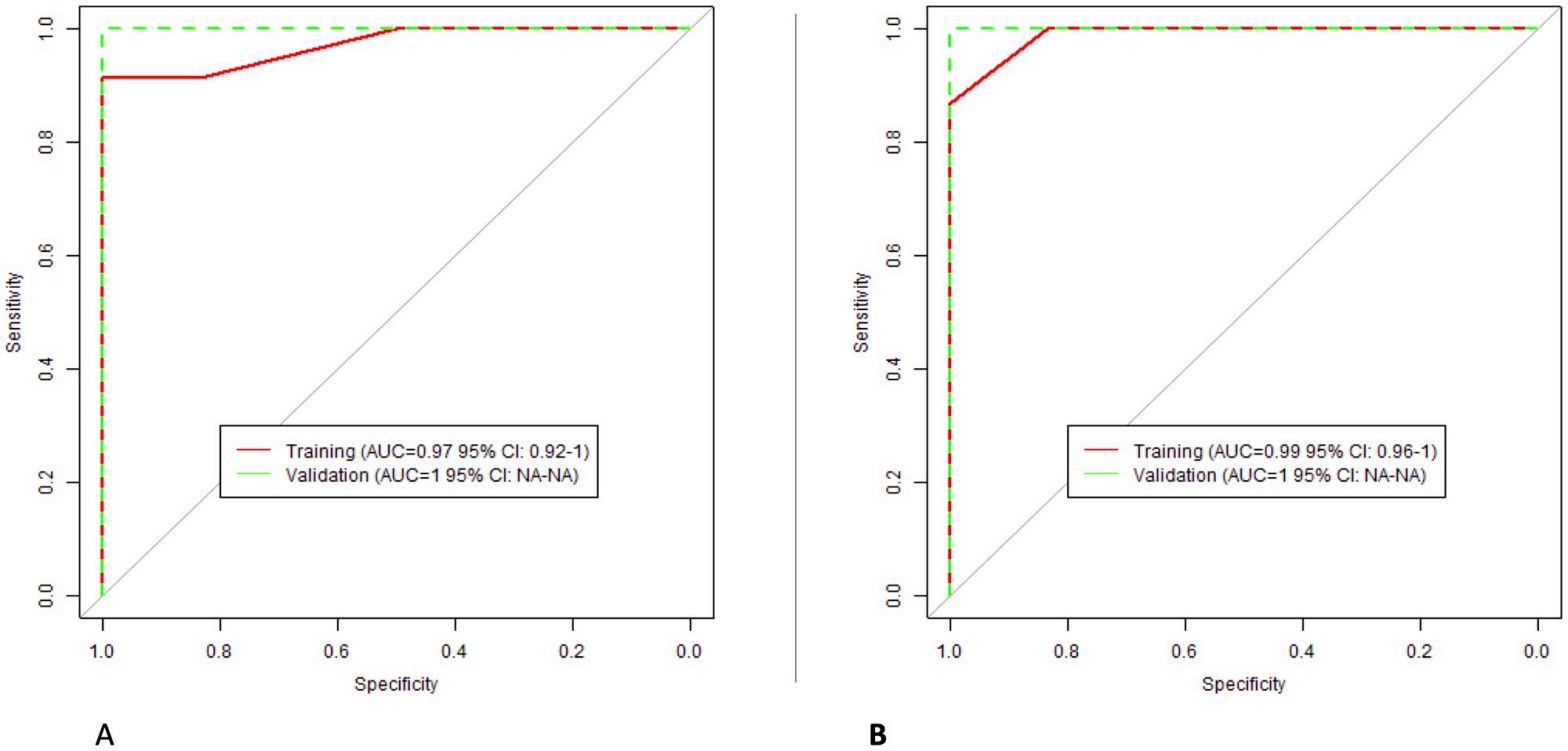
The performance of L-AP classification models; (A) Radiologist 1; (B) Radiologist 2.

For radiologist 1, the selected RFs were: “Gabor Max Z”, and “Gabor sum Z”. For radiologists 2, the selected RFs were: “LoG Z Entropy”, “LoG Z Uniformity”, and “LoG Z MGI”. The performance of the models is presented in Fig 7.

#### Classification models: PVP

**Fig 8:**
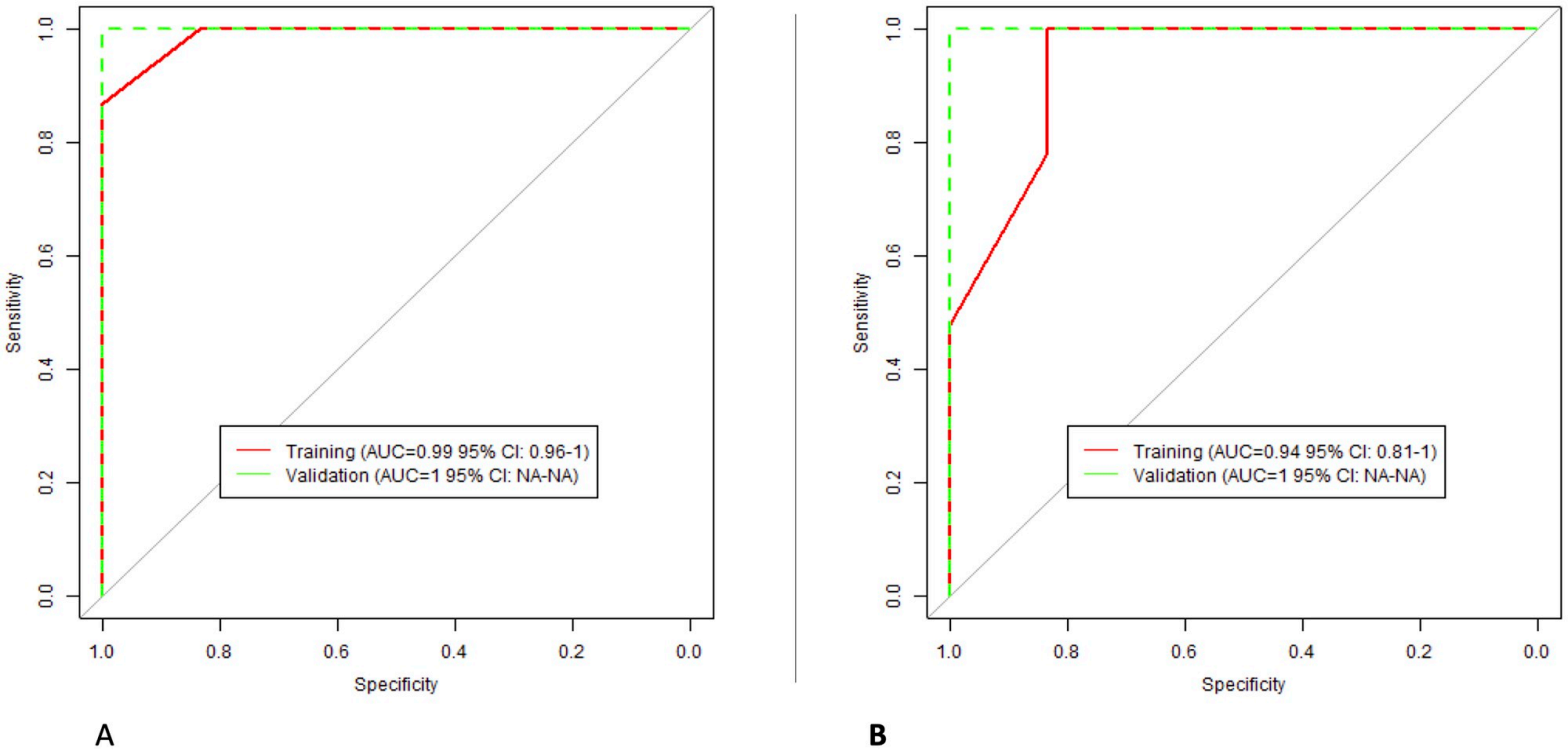
The performance of PVP classification models; (A) Radiologist 1; (B) Radiologist 2.

For radiologist 1, the selected RFs were: “DWF Z L”, “DWF Z LL”, and “LoG Z Uniformity”. For radiologists 2, the included RFs were: “Gabor Median Z”, “Gabor sum Z”, and “Gabor Mean Z”. The performance of the models is presented in Fig 8.

## Discussion

In this study, using our HCC DCE-CT dataset, we assessed the effects of interobserver (inter-segmentation) variability on the reproducibility of RFs, as well as the agreement in RF values within each of the three clinically used imaging phases. Uniquely, we analyzed DCE-CT scans, which provide sequential CT images with specific time intervals (in a range of seconds). Thus, we were able to analyze the reproducibility of RFs within the window of different contrast enhancement phases, which was not previously investigated. As expected, our results showed that the differences in RF values attributed to the variations in imaging timing/sequences were more profound compared to the interobserver effects. At least a quarter of the extracted RFs were reproducible between the two radiologists across different phases, while the number of reproducible RFs for the same radiologist varied between 1% and 37% depending on the pairs of DCE-CT sequences compared. The removal of the highly correlated RFs further significantly reduced the number of reproducible RFs. Henceforth, the segmentation and timing variabilities are important factors that significantly affect the reproducibility of RFs. These findings align with previous studies that assessed the effects of inter-observer variability and clinically used imaging phases on the reproducibility of RFs [13, 15, 36–40].

The findings of this study are consistent with previous research that, in a more limited manner, investigated the effects of contrast enhancement on the reproducibility of RFs [27, 40, 41]. A prior study investigating the reproducibility of HCC RFs across the imaging phases (arterial and PVP) reported that 25% of the original RFs extracted with Pyradiomics toolbox were reproducible [15]. Another published study examining the effects of variations in imaging phase on the reproducibility and predictive power of renal cell carcinoma RFs across the NCE, AP, and PVP scans reported a maximum agreement of 22.4% between the NCE and PVP scans, while the PVP RFs were found to be the least predictive of overall survival in renal cell carcinoma patients [24]. Based on the findings of these studies, the tumor type and site variations also impact the effects of contrast enhancement on the reproducibility of RFs, as different sets of features were reported across studies that investigated different tumor types and sites, in addition to the differences in type and make of imaging hardware.

Unlike prior studies, our data allowed us to investigate within-phase reproducibility. Our results demonstrate that even subtle changes in acquisition time can significantly affect the reproducibility of the extracted RFs. Different phases are acquired to study tumor changes, such as intensity wash-in/wash-out, and they should be analyzed separately in quantitative image analysis. Yet, these were sometimes analyzed together in the literature. The majority of the RFs in this experiment had a very narrow window of reproducibility across the DCT sequences. This confirms the need for both care and caution when investigating RFs acquired in even slightly different contrast enhancement. This is critical for radiomics analyses since most imaging cohorts, whether publicly or privately available, are acquired in different contrast enhancement phases. We strongly recommend the inclusion of a phase determination step in radiomics studies analyzing contrast-enhanced imaging datasets.

Interestingly, our results showed that the numbers of reproducible RFs within each phase and across all pairs differed per radiologist. The highest number of reproducible RFs was observed in the PVP comparisons for both radiologists. While this result could be due to the different numbers of comparisons available for each phase, it might also relate to the appearance of HCC lesions in different imaging phases. Nevertheless, when considering the agreement between radiologists, the L-AP phase had the highest number of reproducible non-correlated RFs, which was also the phase where radiologists performed the first segmentation. It is worth noting that while these RFs have the highest reproducibility, their predictive value must also be considered when selecting the most suitable phase for HCC radiomic studies. Our results reiterate the need for proper quality and reproducibility assessments before performing radiomics analyses.

When considering interobserver variability, our analysis revealed a high agreement in RF values between radiologists in less than a third of the extracted RFs. The number of RFs varied slightly when each phase was assessed separately, with PVP segmentations showing the highest number of reproducible RFs. A similar pattern was observed for intra-phase variability; the highest concordance in RF values was observed across the PVP comparisons.

The evaluation of reproducible RFs within each phase, and for each radiologist, demonstrated a high discriminative ability between the degrees of HCC differentiation in our dataset. These RFs, which intuitively describe the texture of the lesions, thus meet both key criteria for biomarkers: reproducibility and predictivity.

While we carefully designed and executed the statistical analyses in this study, several limitations remain. First, the number of sequences per phase varied among the included patients, which we addressed by standardizing the number of sequences per patient for imaging phase analyses. The scans were selected based on their position within the phase sequences. The different number of within-phase comparisons most likely affects the final number of reproducible RFs per phase. Second, different vendors and imaging parameters were used to acquire the scans, which impacts the reproducibility of RFs. Although the comparisons in this experiment were longitudinal, the rank of patients could be variably affected, ultimately impacting the calculated CCC values. The lack of data prevented the analysis of the effects of variations in imaging acquisition and reconstruction parameters on RFs. While the number of patients included in this study was limited to 39, CCC values are robust in a sample size as small as 10 patients. In addition, previous studies investigating the reproducibility of RFs used a similar number of patients [10, 28, 42–46], including studies on HCC radiomics [47–51]. Lastly, although the reproducible RFs were found to be predictive of the degree of HCC differentiation, the limited number of patients constrains the generalizability of this finding. However, this study serves as a pilot, especially since previous radiomics studies investigating the association between RFs and HCC differentiation have primarily focused on magnetic resonance imaging features.

In conclusion, our results indicate that the majority of RFs are sensitive to variations in the time of acquisition following the injection of a contrast agent. Future radiomics studies should analyze scans acquired in different contrast enhancement phases separately or at least consider the imaging phase during analysis. Furthermore, interobserver variability significantly affects the reproducibility of RFs and must be accounted for in multi-observer radiomics studies. While portal venous phase scans yielded the highest reproducibility within and among radiologists and could be recommended for multi-institutional HCC radiomics studies, biological intent must also be considered when designing such a study.

## Supporting information

S1 File (CSV)
